# Development of a workflow for the selection, identification and optimization of lactic acid bacteria with high γ-aminobutyric acid production

**DOI:** 10.1038/s41598-023-40808-z

**Published:** 2023-08-22

**Authors:** Ateequr Rehman, Giulio Di Benedetto, Julia K. Bird, Valentina Dabene, Lisa Vadakumchery, Ali May, Ghislain Schyns, Wilbert Sybesma, Tim N. Mak

**Affiliations:** 1 dsm-firmenich, Kaiseraugst, Switzerland; 2 FGen/Ginkgo Bioworks, Basel, Switzerland; 3 Bird Scientific Writing, Wassenaar, The Netherlands; 4 Institute of Microbiology, Eidgenössische Technische Hochschule (ETH) Zürich, Zurich, Switzerland; 5 dsm-firmenich, Biodata and Translational Sciences, Delft, The Netherlands; 6 Microbiome Solutions GmbH, Münsingen, Switzerland

**Keywords:** Biological techniques, Biotechnology, Microbiology, Molecular biology

## Abstract

Lactic acid bacteria produce γ-aminobutyric acid (GABA) as an acid stress response. GABA is a neurotransmitter that may improve sleep and resilience to mental stress. This study focused on the selection, identification and optimization of a bacterial strain with high GABA production, for development as a probiotic supplement. The scientific literature and an industry database were searched for probiotics and potential GABA producers. In silico screening was conducted to identify genes involved in GABA production. Subsequently, 17 candidates were screened for in vitro GABA production using thin layer chromatography, which identified three candidate probiotic strains *Levilactobacillus brevis* DSM 20054, *Lactococcus lactis* DS75843and *Bifidobacterium adolescentis* DSM 24849 as producing GABA. Two biosensors capable of detecting GABA were developed: 1. a transcription factor-based biosensor characterized by the interaction with the transcriptional regulator GabR was developed in *Corynebacterium glutamicum*; and 2. a growth factor-based biosensor was built in *Escherichia coli*, which used auxotrophic complementation by expressing 4-aminobutyrate transaminase (GABA-T) that transfers the GABA amino group to pyruvate, hereby forming alanine. Consequently, the feasibility of developing a workflow based on co-culture with producer strains and a biosensor was tested. The three GABA producers were identified and the biosensors were encapsulated in nanoliter reactors (NLRs) as alginate beads in defined gut-like conditions. The *E. coli* growth factor-based biosensor was able to detect changes in GABA concentrations in liquid culture and under gut-like conditions. *L. brevis* and *L. lactis* were successfully encapsulated in the NLRs and showed growth under miniaturized intestinal conditions.

## Introduction

Γ -aminobutyric acid (GABA) is the main inhibitory neurotransmitter in the adult human brain, and that of other vertebrates^1^. GABA production has been documented in a wide range of lactic acid bacteria^2–13^, for which GABA is the end product of the decarboxylation of glutamic acid^10^. Epidemiological evidence has found links between GABA-producing microbes and mood-related conditions^14–16^. GABA is sold in many countries as a dietary supplement that has a calming effect to reduce the negative effects of stress and to improve sleep. A recent review found low to moderate evidence of efficacy for these two parameters, although there were few studies available^17^.

Microbial biosynthesis is regarded as an efficient means of producing GABA^18,19^. Fermented foods containing GABA produced by lactic acid bacteria have been developed^20^, including fermented soy, fruit juice and kimchi^2,8,21–24^. In addition, several species of human-derived lactobacilli and bifidobacteria were shown to produce GABA under simulated intestinal conditions^2^ and in rodents^4^. Therefore, there is interest to develop probiotics that can produce GABA via various food vehicles or directly in the gut. Due to the short half life of GABA (t1/2 is approximately 5 h, with no evidence of accumulation from a repeated dosage regimen of three times per day^25^), the development of active probiotics producing GABA in the gut can potentially increase circulating GABA concentrations without the need for frequent dosing.

Probiotics are live microbes that when administered in adequate amounts confer a health benefit on the host^26^. In our intended application, the probiotic microorganism would be ingested, after which it would produce GABA in the intestinal tract. The probiotic must have a history of safe use in humans, and it must also be able to grow in the large intestine, where it can produce GABA. Therefore, screening and strain improvement programs should ideally select microorganisms that meet these criteria.

Classical strain improvement techniques used together with newer approaches such as microbial genomics and cell engineering allow complex phenotypic traits to be combined, which may be a useful strategy to identify novel probiotic strains^27,28^. Techniques such as miniaturization allow the development of efficient workflows that can process large strain libraries arising from mutagenesis and identify mutants with multiple characteristics. For this study, the ability to survive passage through the digestive tract to the large intestine, and GABA production were concurrent characteristics required for candidate strains.

A difficulty in selecting strains capable of surviving passage in the human digestive tract is that the human gut environment is complex. It can be challenging to create conditions in a high throughput screening program that can adequately predict survival and growth in vivo. Even so, it is important to consider growth and production in the final application because it ultimately affects the success of the project in identifying a probiotic that has a relaxing effect through GABA production in the intestinal tract. The Simulator of the Human Intestinal Microbial Ecosystem (SHIME) provides a validated, modulated system for testing in the intestinal tract. It has been used widely in human microbiome projects as an appropriate medium for testing growth under gut-like conditions^29,30^.

In terms of selecting strains with high GABA production, biosensors allow the direct measurement of macromolecules with a high selectivity. However, they usually require the use of recombinant techniques that are not desirable in microorganisms used for human consumption. The use of co-culture with a probiotic strain variant and whole-cell biosensor could allow the identification of probiotic strains with desirable phenotypes, especially in the case of secreted products. Techniques such as miniaturization allow the development of efficient workflows that can process large strain libraries arising from mutagenesis and identify, in a high throughput manner, mutants with favorable characteristics. As an example, hydrogel microcarriers of nanoliter volume have been successfully applied for the high-throughput screening of natural or recombinant strains^31^. In the case of this project, the desired improved probiotic strain should show high production levels of GABA and good viability in the digestive tract, where it should exert its function.

This research study had three main aims. First, to identify potential probiotic strains capable of producing GABA that can be used as a chassis for classical strain improvement. Secondly, to develop an efficient methodological workflow to select GABA-overproducers from a library of mutants. And thirdly, to test the survival, growth and GABA production of the selected strains in a simulated intestinal environment.

## Results

### Identification of GABA producers

The initial literature research and industry strain collection screening identified 402 bacterial strains that could be developed as probiotics. Genomic data was not available for 34 of these strains, which were excluded. After the in silico screening, a further 128 were excluded from the study as as no genes for GABA production were identified. After grouping the strains into 13 taxonomic classification groups, at least one strain from each group was selected. A taxonomy study was performed for two strains that could not be found in any public strain collection. Two strains showing the same taxonomic properties were identified that were available from a strain collection.

**Figure 1:**
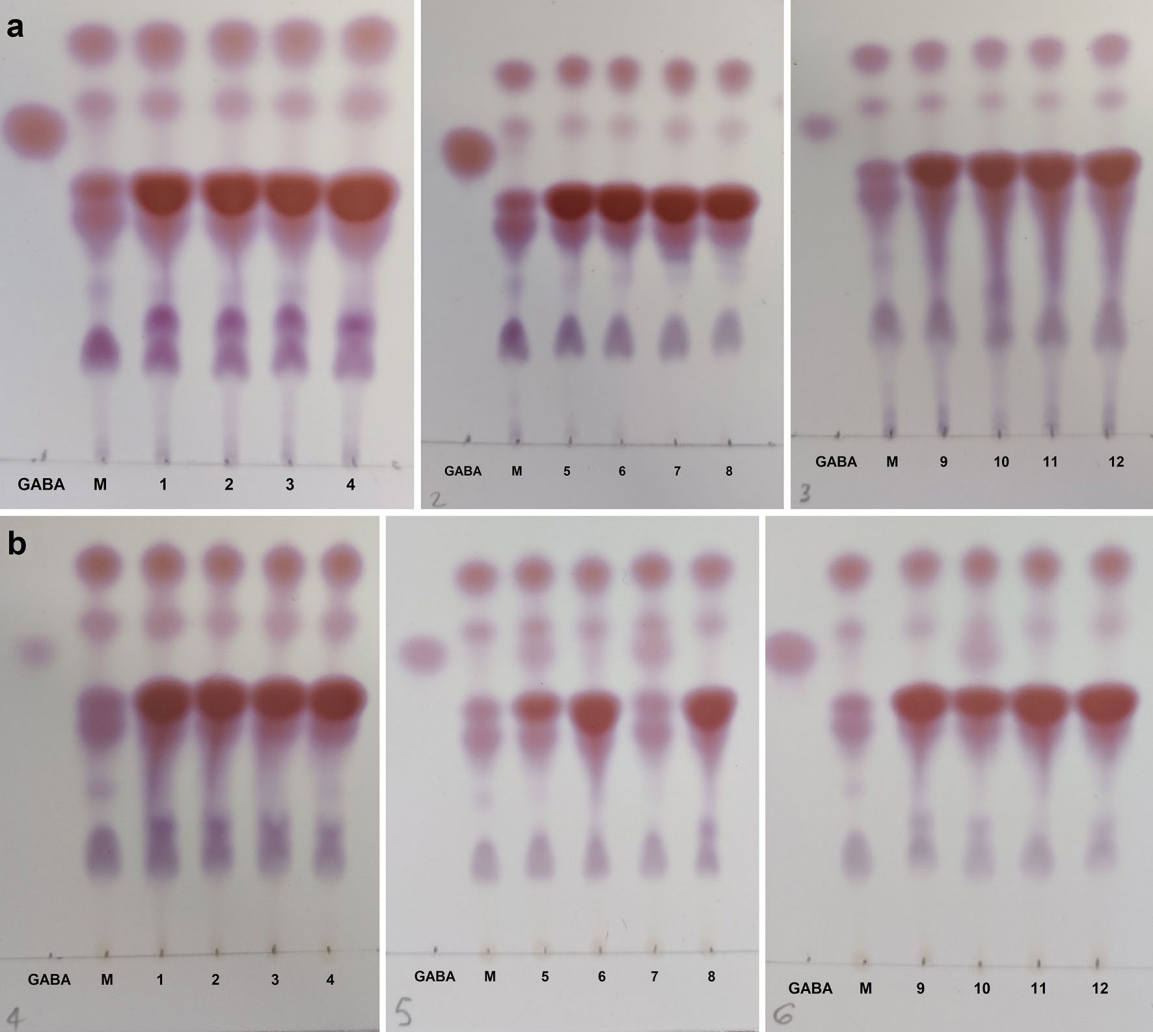
Evaluation of GABA production in supernatants from BHI and MRS cultures. TLC sheets with 1μL supernatants isolated from (**a**) BHI cultures, and (**b**) MRS cultures of after 75 h cultivation. 1μL of GABA 10 g/L in distilled water (lane ‘GABA’) represents the positive control, and the negative control is represented by the lane ‘M’, which corresponds to 1μL of culture medium. Strains: (1) *B. animalis subsp. lactis* BB121, (2) *L. rhamnosus* GG, (3) *L. helveticus* R00522, (4) *B. coagulans* MTCC 5856, (5) *L. brevis* DSM 20,054, (6) *L. lactis* CSK-C123, (7) *L. lactis* DS75843, (8) *S. thermophilus* ST-105, (9) *L. acidophilus* LA-5, (10) *B. adolescentis* DSM 24,849, (11) *S. thermophilus* 682A, (12) *B. longum* R01752.

The 17 potential GABA producers selected for GABA screening are described in Table 1. The three *Lactococcus cremoris* strains showed no growth due to a prerequisite for lactose and therefore were excluded from further testing. The results of the thin layer chromatography (TLC) analysis of supernatants from both media are displayed in Fig. 1 for 12 strains. GABA production was found for *L. lactis* DS75843, *L. brevis* DSM 20054, and *B. adolescentis* DSM 24849 in De Man, Rogosa and Sharpe (MRS) medium. GABA production was confirmed in two biological replicates from each strain. The amount of GABA measured by HPLC was 2.94, 2.56 and 1.71 g/L for *L. lactis* DS75843, *L. brevis* DSM 20054 and *B. adolescentis* DSM 24849, respectively.

**Table 1:** Description of strains selected and cultivation conditions.

Strain	Strain number	Obtained from	Resuspension buffer	Solid medium	Cultivation conditions
1	*Bifidobacterium animalis* subsp. *lactis* BB-12	Commercial probiotic	Mitsuoka buffer	M58 agar	Anaerobic
2	*Lactobacillus rhamnosus *GG	Commercial probiotic	Mitsuoka buffer	MRS agar	Aerobic
3	*Lactobacillus acidophilus* LA-5	Commercial probiotic	Mitsuoka buffer	MRS agar	Aerobic
4	*Bifidobacterium longum* R0175	Commercial probiotic	Mitsuoka buffer	M58 agar	Anaerobic
5	*Lactobacillus helveticus* R0052	Commercial probiotic	Mitsuoka buffer	MRS agar	Aerobic
6	*Bacillus coagulans* MTCC 5856	Commercial probiotic	Peptone buffered water	2xTY	Aerobic
7	*Levilactobacillus brevis* DSM 20054 (*L. brevis* NPS-QW-145)	DSMZ	Mitsuoka buffer	MRS agar	Aerobic
8	*Bifidobacterium adolescentis* DSM 24849 (*B. adolescentis* 150)	DSMZ	Mitsuoka buffer	M58 agar	Anaerobic
9	*Lactococcus lactis* DS84445	dsm-firmenich proprietary strain	None	MRS agar	Anaerobic
10	*Lactococcus cremoris* DS84447	dsm-firmenich proprietary strain	None	Elliker agar	Aerobic
11	*Lactococcus lactis* DS75843	dsm-firmenich proprietary strain	None	MRS agar	Anaerobic
12	*Lactococcus cremoris* DS79469	dsm-firmenich proprietary strain	None	Elliker agar	Aerobic
13	*Lactococcus cremoris* DS79470	dsm-firmenich proprietary strain	None	Elliker agar	Aerobic
14	*Streptococcus thermophilus* DS67400	dsm-firmenich proprietary strain	None	MRS agar	Anaerobic
15	*Streptococcus thermophilus* DS77530	dsm-firmenich proprietary strain	None	MRS agar	Anaerobic
16	*Lactobacillus plantarum* DS82101	dsm-firmenich proprietary strain	None	MRS agar	Anaerobic
17	*Lactobacillus plantarum* PS128	Commercial probiotic	Mitsuoka buffer	MRS agar	Anaerobic

### Transcription factor-based biosensor

**Figure 2:**
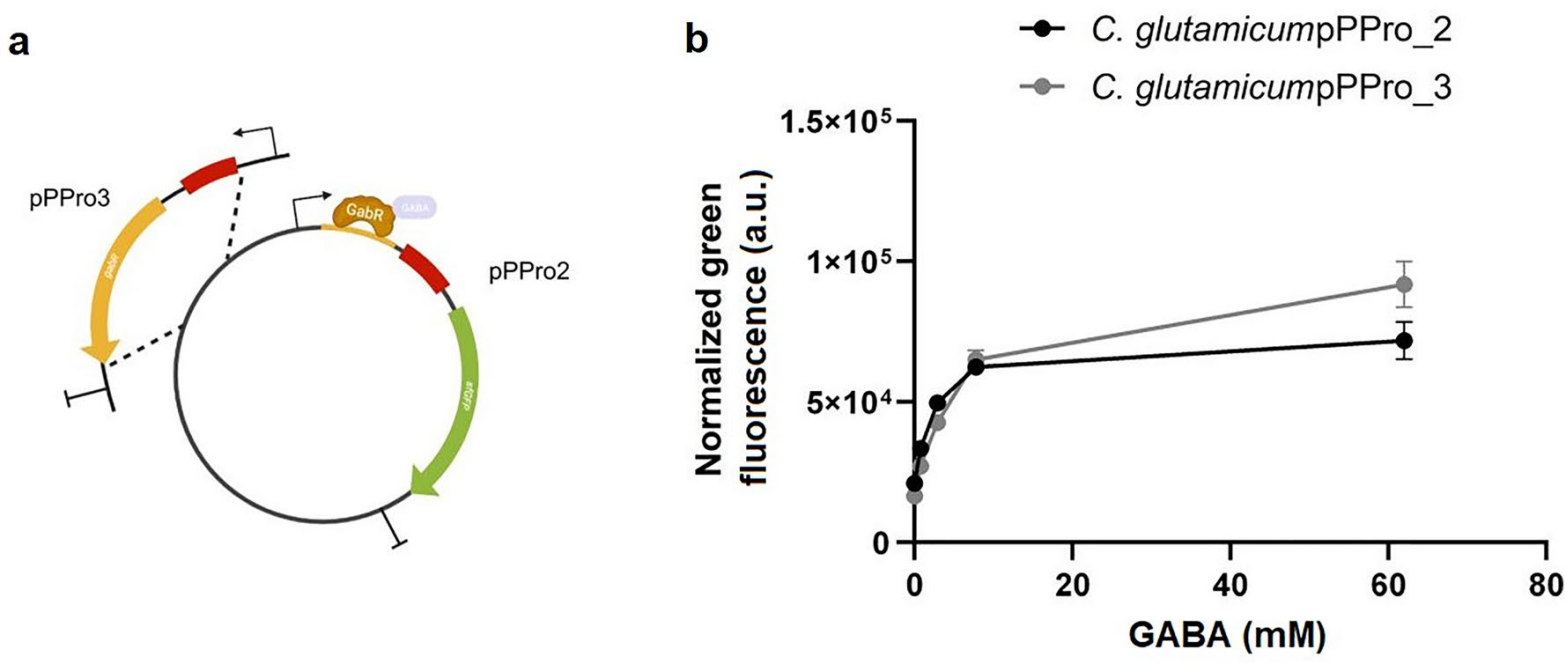
Transcription factor-based sensor design. Panel **a**: Plasmid maps of the two transcription factor-based sensors. pPPro2 harbors the binding region of GabR (yellow line), the ribosome binding site (red box) and *sfGFP* gene (green arrow). pPPro3 harbors additionally the *GabR* gene (yellow arrow). Panel **b**: Dot plot with the values of green fluorescence normalized by optical density plotted against the different concentration of externally added GABA in the liquid cultures (black line for the *C. glutamicum* carrying pPPro2, grey line for the strain with pPPro3. Error bars represent the error calculated based on two biological replicates.

The two constructs ([1] upstream of gabT start codon, pPPro2; [2] with the bidirectional gabTDP promoter, pPPro3) were cloned into *C. glutamicum* and tested in liquid culture. Figure 2 shows the plasmid maps of the two constructs (Panel A). In liquid culture, both constructs showed similar results in the presence of GABA, with a dynamic range between 0 and 8 mM GABA and > 2.9-fold difference in intensity upon the addition of saturating concentrations of GABA (Panel B).

**Figure 3:**
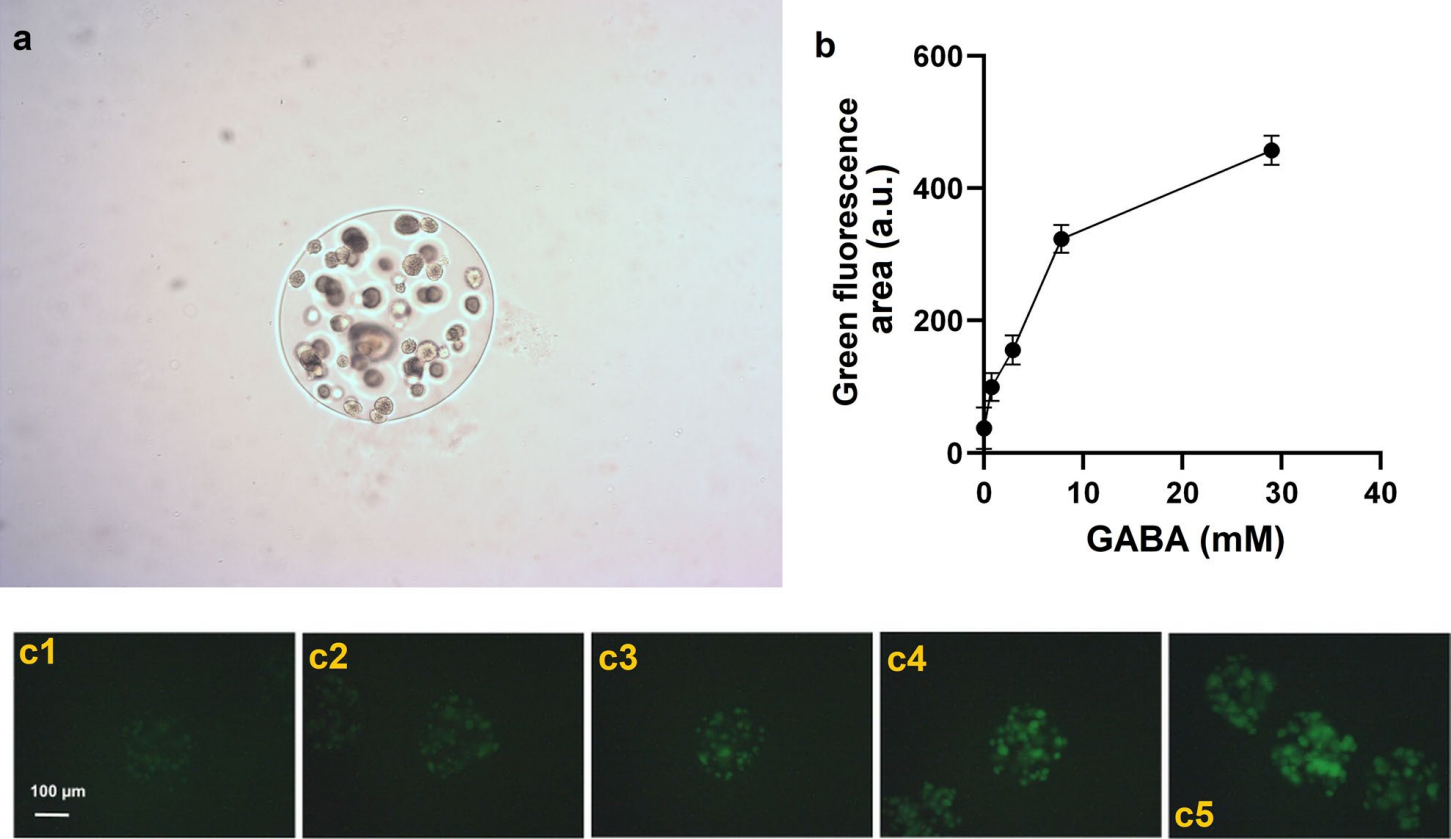
Transcription factor-based biosensor in NLRs. Panel **a**: brightfield microscopy of NLRs harboring the transcription factor-based biosensor. Panel **b**: Response curve of the transcription factor-based biosensor in NLRs upon addition of different concentrations of GABA. The values plotted represent the mean of the integrated green fluorescence measured with flow cytometry, while the error bars represent the standard deviation of the 2983, 3393, 2962, 2912, 3008 events recorded for each concetration tested (0–3 g/L GABA). Panel **c**: green fluorescence microscopy pictures of NLRs harboring the transcription factor-based biosensor (average occupation of 200 cells per NLR). The NLRs were incubated for 24 h at 37 C in 2xTY medium supplemented with (1) 0, (2) 0.08, (3) 0.3, (4) 0.8 and (5) 3 g/L GABA.

After encapsulation in nanoliter reactors (NLRs: see methods for a complete description) and incubation with different GABA concentrations, only the *C. glutamicum* strain with plasmid pPPro2 showed differences in green fluorescence when GABA was added. Figure 3A depicts the NLR under a light microscope and Fig. 3C the average green fluorescence of each NLR with the biosensor containing the upstream promotor after cultivation in medium with varying concentrations of GABA. Flow cytometry data shows a linear correlation between fluorescence intensity and GABA concentration in the medium up to 10 mM and a 12-fold change in intensity recorded with the highest concentration of GABA tested (Fig. 3B).

### Growth factor-based biosensor

**Figure 4:**
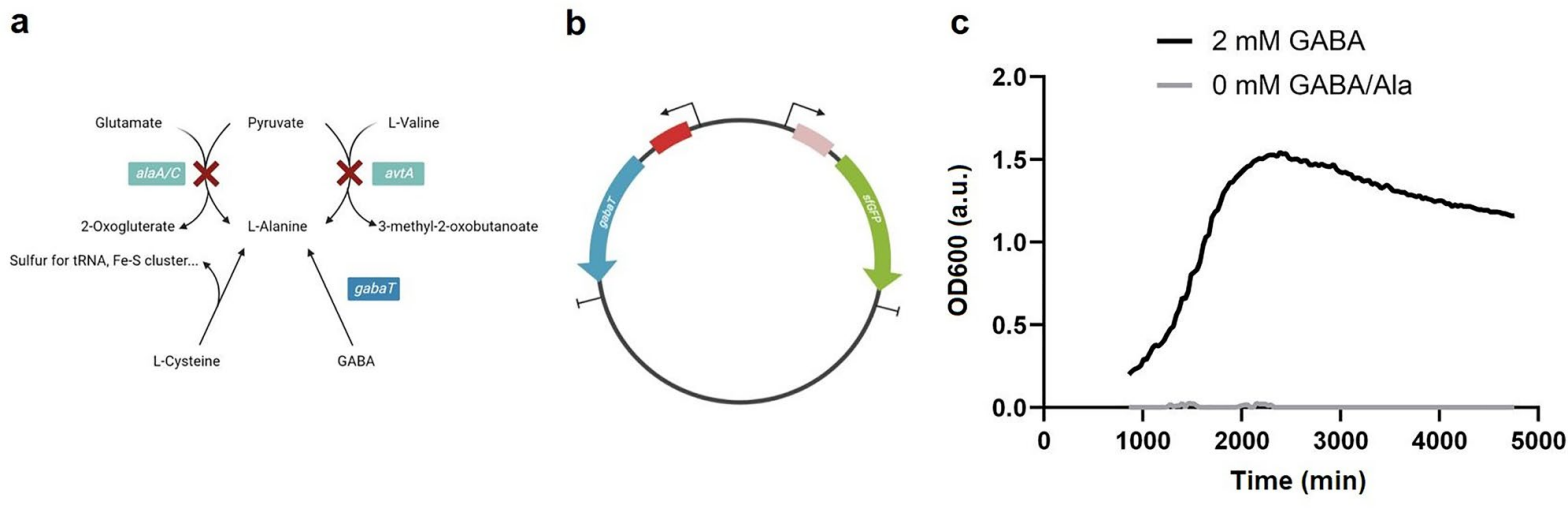
Growth-based biosensor. Panel **a**: Schematic drawing of the alanine metabolism in *E. coli*. Pyruvate can be converted over two pathways with enzymes encoded either by *alaA/C* or *avtA* to L-alanine. In the growth-based sensor these genes are knocked out and another gene *gabaT* is introduced to convert GABA into alanine. Panel **b**: The plasmid harboring *gabaT*: the red boxes represent RBS with various binding strength: red, weak binding; pink, strong binding. The blue arrow represents *gabaT* and the green arrow represents *sfGFP*. Panel **c**: Growth curve of the growth-based sensor in presence and absence of 2 mM GABA. The curve represents the mean value of 3 biological replicates.

The growth factor-based biosensor, based on auxotrophic complementation whereby 4-aminobutyrate transaminase (GABA-T) transfers the GABA amino group to pyruvate, thus forming alanine in an alanine auxotroph, was initially tested in liquid culture in the presence and absence of the target molecule. The strain shows no visible growth in the absence of GABA. In contrast, the growth is recovered when 2 mM of target molecule is added (Fig. 4, panel C), indicating that it could detect GABA in the supernatant of producer strains.

**Figure 5:**
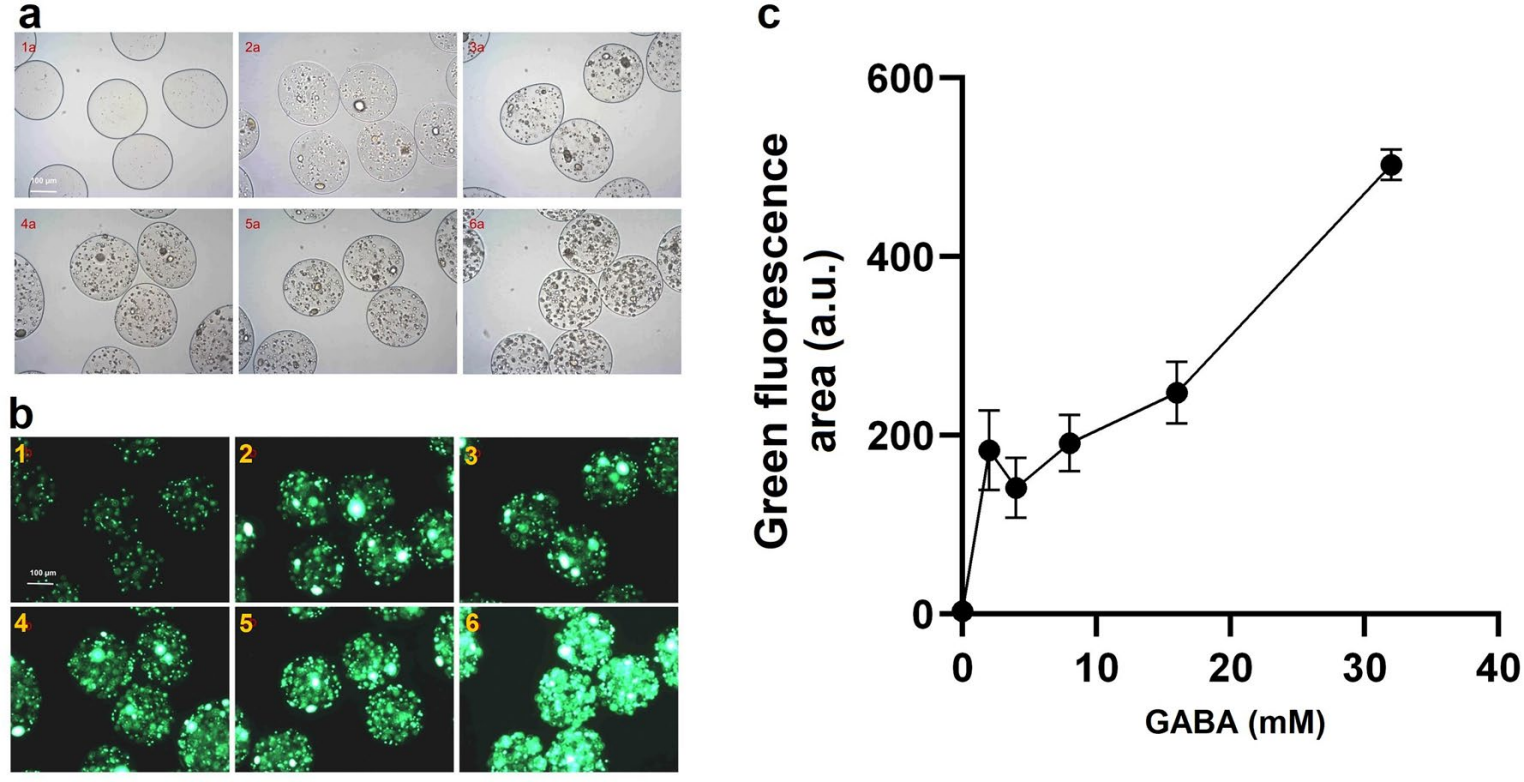
Improved growth-based sensor in NLRs. Panel **a**: Brightfield and **b**: green fluorescence microscopy pictures of NLRs harboring the improved growth-bases biosensor (average occupation of 200 cells/NLR). The NLRs were incubated for 2 days at 37 °C in M9 medium supplemented with (1) 0, (2) 2, (3) 4, (4) 8, (5) 16 and (6) 32 mM GABA. Exposure time for (2–6) was 100 ms and for (1) 1 s. Panel **c**: Response curve of the growth-based biosensor in NLRs upon addition of different concentrations of GABA. The values plotted represent the mean of the integrated green fluorescence measured with flow cytometry, while the error bars represent the standard deviation of the 1667, 1887, 1024, 993, 1360, 1587 events recorded for each concentration (0–32 mM GABA).

The *E. coli* biosensor was encapsulated and grown in M9 medium, which is chemically defined and does not contain alanine, at varying concentrations of GABA (M9 medium 10 × solution: 128 g/L Na_2_HPO4*7H_2_O, 30 g/L KH_2_PO_4_, 5 g/L NaCl, 10 g/L NH_4_Cl). Although a correlation was seen between growth and GABA concentration, the sensitivity and dynamic range were poor (results not shown). Therefore, an improved growth factor-based biosensor was created by adjusting the expression of the transaminase; surprisingly, a weaker RBS upstream of GABAT resulted in a better sensitivity and dynamic range (Fig. 5). The optimized biosensor was more sensitive, showing a 44-fold difference between the green fluorescence intensities measured with no external GABA or a concentration of 4 mM of the target molecule. In addition, the dynamic range was also improved with a significant increase in green fluorescence even at 32 mM GABA.

### Cultivation of GABA producers and biosensors, and growth under gut-like conditions

The GABA producers and biosensors were encapsulated separately and cultivated in static and anaerobic conditions for up to 5 days to test for growth and performance after encapsulation, and to test growth under gut-like conditions (see Table 2).

**Table 2:** Cultivation conditions of the nanoliter reactors.

NLR type	Vessel type	Washing solution	Medium	Incubation conditions	Comment
GABA-producers	100 ml sterile plastic container	Washing buffer: 10 mM CaCl_2_, 10 mM MES pH6, 0.05% cysteine	MRS or SHIME	Anaerobic, static, 37ºC, up to 96 h	–
Transcription factor-based biosensor	Sterile 50 ml shake flask	10 mM CaCl_2_	2xYT with with 5 mM CaCl_2_	Aerobic, 37 °C, 250 rpm, up to 45 h	Several flasks were prepared with different final concentrations of GABA (between 0 and 62 mM)
Growth factor-based biosensor	Sterile 50 ml shake flask	Washing buffer	M9 (alanine-free)	Aerobic, 37 °C, 250 rpm, up to 45 h	Several flasks were prepared having different final concentrations of GABA (between 0 and 32 mM). A positive control was included with 2 mM alanine instead of GABA
Gut-like conditions	15 ml Falcon tubes	Washing buffer	SHIME	Micro- and an-aerobic, 37 °C, up to 45 h	Different cultivation conditions were tested to ensure proper growth of the strain and good mixing of the turbid medium

**Figure 6:**
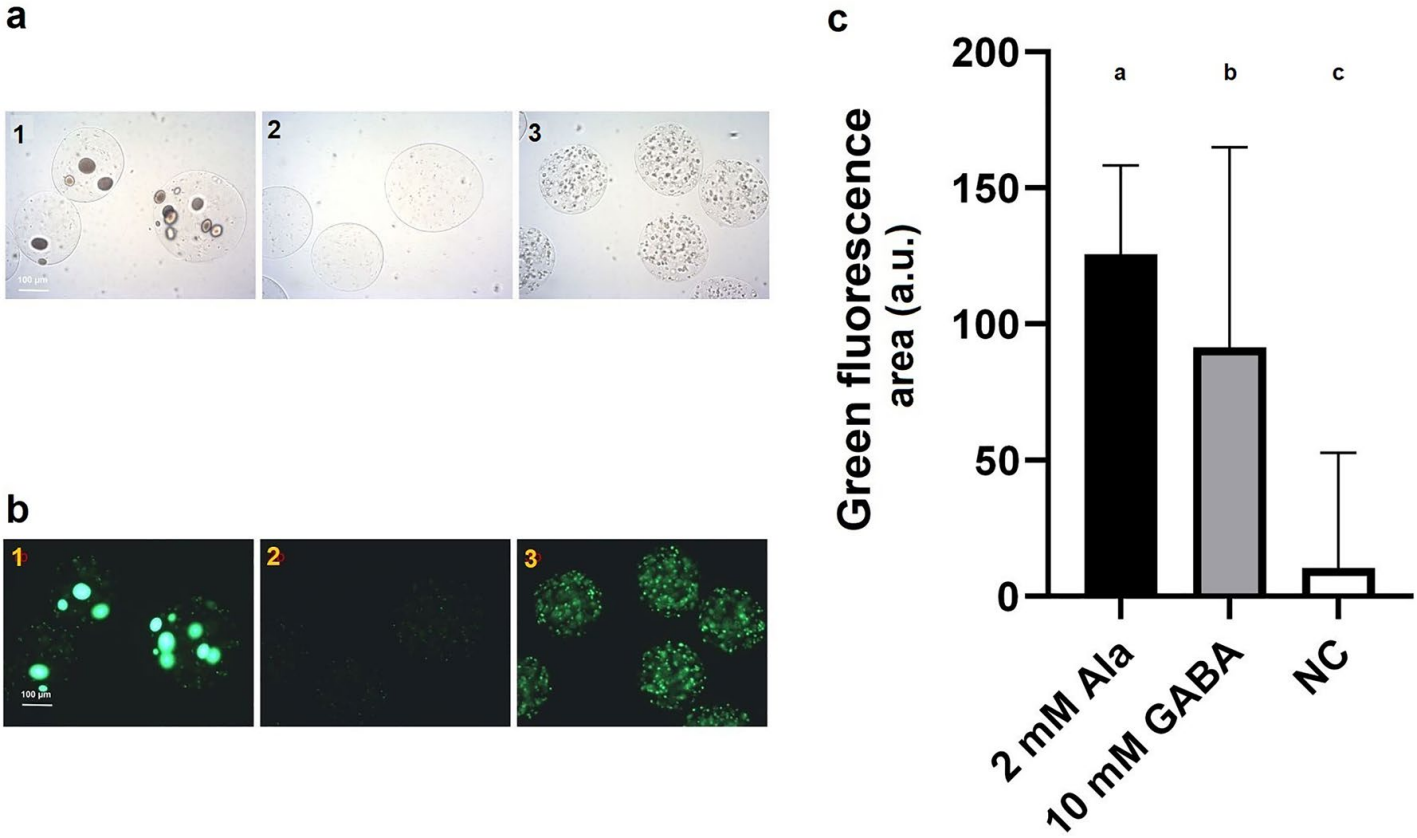
Improved growth-based biosensor in NLRs in screening conditions. Left panels: (**a**) brightfield and (**b**) green fluorescence microscopy pictures (exposure time of 50 ms) of NLRs harboring the improved growth-based sensor. The NLRs were incubated for 3 days at 37 C in modified SHIME. The media was supplemented with either (1) 10 mM GABA or (3) 2 mM alanine. (2) represents the sample with SHIME medium only. Panel **c**: Green fluorescence of the growth-based biosensor in NLRs in SHIME medium with 2 mM alanine, 10 mM GABA or no additional molecule added (i.e. negative control, NC). The mean values of the integrated green fluorescence measured with flow cytometry are respectively: 126, 91 and 10 a.u.. The error bars represent the standard deviation of the 1268, 1245, 1257 events measured for the three samples cultivated with 2 mM Ala, 10 mM GABA and SHIME medium, respectively. The standard deviation values are 33, 73 and 42 a.u. Different letters denote significant differences at *p* < 0.001.

Positive biomass formation in the NLRs was detected for the producer strains *L. lactis* DS75843 and *L. brevis* DSM 20054, however growth was very poor for *B. adolescentis* DSM 24849. When the *C. glutamicum* based biosensor with the upstream promotor was tested in SHIME medium with a low oxygen content, no growth was detected. The optimized *E. coli* growth factor-based biosensor was tested under gut-like conditions. When the tube was completely filled with medium (anaerobic), no growth was seen. When the amount of available oxygen was increased by half-filling tubes (aerobic), growth was observed and the biosensors could detect externally-added GABA. Under these conditions, differences in the intensities of green fluorescence of the samples with GABA or alanine added were seen, compared to the negative control in which the auxotrophy was not recovered (Fig. 6).

## Discussion

The aim of this project was to identify a probiotic strain able to produce GABA and develop a workflow aiming to increase GABA production via a high-throughput classical strain improvement program. The initial literature review identified a large number of candidate probiotic strains that were potential GABA producers and had genomic data available. The in silico analysis identified 17 candidate strains for further testing. The in silico analysis screened for the presence genes for the two isoforms of the glutamate decarboxylase (*gadA* and *gadB*), which converts glutamate to GABA. Two or more genes for glutamate decarboxylase can coexist in one strain, which may be a desirable characteristic: glutamate decarboxylases from different probiotics have different pH optima^10^, thus the extended range in which the GABA-producing enzyme is active may be an advantage in the acidic-neutral pH gradient in the human digestive tract^10,32^.

After cultivation in MRS or BHI medium, thin layer chromatography confirmed GABA production in three strains: *L. lactis* DS75843, *L. brevis* DSM 20054 and *B. adolescentis* DSM 24849. The NLR screening platform was selected as an appropriate technique to allow high-throughput screening of a strain library that could be developed based on one of the three GABA producers. Two biosensors were developed for NLR-based co-culture with the purpose to select for strains that produce GABA under intestinal conditions. The first was a transcription factor-based biosensor that relied on the interaction between GABA and the transcriptional regulator GabR and was built in *C. glutamicum*. The second was a growth factor-based biosensor constructed in *E. coli* and was based on auxotrophic complementation, achieved by expressing 4-aminobutyrate transaminase (GABA-T) that transfers the GABA amino group to pyruvate, thus forming alanine.

The growth in NLRs of the three producer strains and two biosensors was tested. The *L. lactis* DS75843 and *L. brevis* DSM 20054 strains produced large colonies in the NLRs, and the growth factor-based biosensor was able to detect GABA in gut-like conditions. This project was able to identify two GABA-producing probiotic strains, and developed a workflow that could be used as a basis for a strain development campaign.

When the potential of strains to produce GABA was initially tested, no strain was able to produce GABA in BHI, while three strains were able to produce GABA in MRS, despite a comparable cell density reached in both media. This clearly indicates that the medium plays a major role in the production of GABA. As GABA production is induced as an acid stress response^12^, the native glutamate decarboxylase enzyme is only active at a low pH^10^^.^ This could provide a plausible explanation as to why GABA could not be detected in the BHI medium that has a pH of approximately 7.4, i, while the pH in MRS isH ≤ 6.2. Strain improvement campaigns need to consider how pH affects both growth and production of GABA by probiotic microorganisms. Therefore, it may be beneficial in the screening phase to test the strains under varying pH conditions. This can be achieved by adjusting the buffering capacity of the medium, or using CO_2_/HCO_3_-buffering in a CO_2_-rich atmosphere to maintain the intended pH in miniaturized cultivation conditions^33^, to gain a better understanding of how the strain will perform in manufacturing and its final use.

Incorporation of *B. adolescentis* DSM 24849 in NLR in either SHIME medium or rich media did not result in cell growth required for the selection approach. It is assumed that exposure to oxygen for 30 min during the NLR preparation affected the fitness of the cells.

When testing the transcription factor-based biosensor in NLRs, the strain harboring the plasmid pPPro2 was sensitive to the concentration of GABA as initially measured in liquid culture. However the version containing the entire *gabR* gene (pPPro3) did not show any significant difference in the intensity of green fluorescence when GABA was added. These results were surprising and would need further investigation to better understand the regulation mechanism of the gabTDP operon in *C. glutamicum* when GabR is overexpressed in different cultivation conditions.

A further consideration in developing a workflow for a co-culture with biosensors is the divergent growth conditions required by the producer strains compared to the biosensors. In this series of experiments, the transcription factor-based biosensor in *C. glutamicum* did not grow under low oxygen conditions. Even though *C. glutamicum* is a robust facultative anaerobe, biomass production under low oxygen conditions is poor and the shift to anaerobic growth results in a profound shift in metabolism^34^. On the other hand, the *E. coli*-based biosensor showed adequate growth under microaerobic conditions, and expected differences in fluorescence were seen when GABA or alanine were added compared to the negative control. These results suggest that the cultivation conditions should be adapted and customized for each producer-biosensor co-cultivation, taking into account the oxygen requirements for growth and production.

This project sets the basis for a strain development campaign to improve the production of GABA. Screening was performed under conditions that simulate the small and large intestine, which increases the likelihood that a probiotic strain can be identified that is able to produce GABA in the intestinal tract when the probiotic is consumed as a supplement or in a fermented food.

## Methods

### Identification of potential GABA producers

To identify potential GABA producers, literature research and in silico screening of commercially available strains and an industrial strain collection was undertaken with the following criteria:

1. A known probiotic
2. Capable of growth in the human intestinal tract
3. Containing the ability to synthesize GABA
4. Accessible genomic information

A database of genes relevant to the GABA pathway was constructed by searching the UniProt database^35^ with the following query:

(gene:gada OR gene:gadb OR gene:gadc OR gene:gabt OR gene:gadr OR gene:gabD) (taxonomy:"Bifidobacterium [1678]" OR taxonomy:"Lactobacillus [1578]" OR organism:"Streptococcus thermophilus [1308]" OR taxonomy:"Lactococcus lactis [1358]" OR taxonomy:"Bacillus coagulans [1398]" OR taxonomy:"Leuconostoc [1243]" OR taxonomy:"Propionibacterium [1743]")

The taxonomy terms in the query correspond to species and genera of strains and species identified in the industrial portfolio and commercially available probiotics. The query resulted in the identification of 214 gene seuqences.

Next, the genomes of strains with available genomic data were annotated using Prokka v1.14.6 using default parameters. The resulting proteome (amino acid) sequences aligned against the custom GABA database described above using USEARCH v11.0.667 (‘ublast’ option, min. alignment identity = 70%, min. target and query coverage = 70%) in a BLAST-like in silico screening to check for the presence of two isoforms of glutamate decarboxylase, *gadA* and *gadB*, which are genes required for GABA synthesis. Strains that did not contain these genes were excluded from further analysis. The remaining strains were classified into 13 groups according to species and sequence homology^36^.

### Strain isolation

Strains obtained as lyophilized material were isolated as follows: approximately 2 g was suspended in 2 ml suspension buffer and rehydrated at room temperature for 30 min. The suspension was diluted to a cell concentration of approximately 10^3^ based on the package leaflet concentration. 100 μL was plated onto solid media and incubated at recommended growth conditions for the strains at 30 °C. In the case of a preparation containing more than one strain, different phenotypes were streaked separately on new agar plates until all the colonies showed a similar growth behavior. Strains received from the dsm-firmenich strain collection were glycerol stocks and were directly plated onto solid medium and incubated at recommended growth conditions for the strains (Table 1).

### Cultivation and 16S rRNA sequencing of potential GABA producers

Once growth was visible, up to three colonies for each phenotype were selected and inoculated into 14 ml round-bottomed test tubes containing 12 ml or 3 ml (aerophilic strains) medium. Cultures with *Bacillus coagulans* MTCC 5856 were shaken at 250 rpm (2.5 cm orbit diameter); otherwise strains were cultivated under static conditions at 30 °C until an OD_600_ > 1 was reached (approximately 48 h). This resulting suspension was used to create 20% v/v glycerol stocks, and was also streaked onto solid medium to monitor the phenotype.

3 ml culture (1 ml for the aerophilic strains) was used to isolate genomic DNA with the kit (Wizard Genomic DNA purification kit, Promega). Universal primers (Primer PP9 and Primer PP10) were used to amplify the 16S rRNA gene sequences, and the resulting PCR product purified and sequenced using the same primers. Plasmid sequences are provided in the Supplementary Information Table S1. Alignment of sequencing data was performed using BLASTN against the standard databases and their related genus.

### Thin layer chromatography analysis of supernatant cultures

All strains were cultivated in BHI and MRS medium (Merck) supplemented with 1% w/v glutamate for the initial evaluation of GABA production. These media were chosen after reviewing the literature for the best options for growth of lactic acid bacteria^37–39^. 1:100 diluted glycerol stocks were inoculated into 12 ml or 3 ml medium in a test tube and grown according to recommended cultivation conditions for up to 5 days. Cultures were sampled every 24 h to monitor the growth and isolate supernatant by centrifuging an aliquot at 10,000×g for 30 s. The time period with the highest OD was selected for TLC analysis. The qualitative TLC procedure described by Yogeswara was used^40^. Briefly, 0.5 to 2 μL supernatant was spotted onto a TLC sheet ALUGRAM Xtra SIL G using glass capillaries approximately 1 cm from the bottom and allowed to dry. The TLC sheet was immediately placed in a glass chamber with a mobile phase consisting of 1-butanol: acetic acid: distilled water (5:2:2). Once the mobile phase reached 90% of the height of the silica layer, the slide was isolated from the mobile phase and dried. A solution of 0.5% ninhydrin (w/v) in ethanol was sprayed over the silica. A GABA stock solution was used as a positive control, and 1 μL BHI or MRS medium was used as a negative control. Color formation at the height of the positive control indicated GABA production by the strain. After GABA producers were identified, two biological replicates from each strain were tested in MRS medium after 71 h of cultivation to confirm the initial results. The GABA concentration in this supernatant was measured via HPLC.

### Preparation of transcription factor-based and growth factor-based biosensors

Due to the challenges of co-cultivating strains with different growth requirements, two different approaches were taken in different hosts to increase the chance of successfully developing a biosensor capable of growth under conditions mimicking the intestinal tract. The transcription factor-based design was built in *C. glutamicum* and relied on the interaction between GABA and the transcriptional regulator GabR^12^, which then activates the transcription of a reporter. Two constructs were prepared, one having the reporter plasmid either upstream of the *gabT* start codon and the other containing the entire *gabR* gene (see Fig. 2, panel A). The growth factor-based design is based on the generation of an alanine auxotrophic *E. coli* strain^41^ (see Fig. 4, panels A and B). Both biosensors produced the green fluorescent protein sfGFP as a marker of transcription or growth.

#### Transcription factor-based biosensor in Corynebacterium glutamicum

The transcription factor-based biosensor was developed in *Corynebacterium glytamicum*, which is able to utilize GABA, as described in a previous publication^12^. Two biosensor designs were tested: one was based on the gabR binding region (identified within the gabTDP promoter) upstream of a reporter gene (*sfGFP*), and the bidirectional gabTDP promoter controlling both the *gabR* gene and the *sfGFP* gene. Information about the plasmids used is found in Supplementary Information Table S2.

For the design with the GabR binding region, part of a synthesized DNA fragment (Twist Bioscience) containing the sequence that covers the 500-bp fragment region upstream of *gab*T was used. At the 5’-end of the sequence a *Spe*I site was added, while at the 3’ end a sequence was added corresponding to the first 29 bp of the *sfGFP* gene. For the design with the bidirectional *gabTDP* promoter, the insert was generated using the primers PP11 and PP12 to amplify the whole *gabR* gene and the *gabTDP* promoter directly from the genomic DNA of *C. glutamicum* ATCC 13,032.

The primers were designed to generate a DNA fragment with the same modifications as mentioned above. *sfGFP* was amplified from a plasmid using the primers PP13 and PP14. The primer PP14 was designed to introduce an *Xba*I site at the end of the reporter gene. Assembly PCR was performed using as templates the generated inserts and the primers PP11 and PP14. The two PCR products and a shuttle vector *E. coli*/*C. glutamicum* (pPPro_1) were digested with the enzymes *Spe*I and *Xba*I, followed by ligation and transformation of the ligation mixes into chemically competent DH10B *E. coli* cells. The correct sequence of the generated constructs (pPPro_2 and pPPro_3) was verified by sequencing and subsequently used to transform competent *C. glutamicum* ATCC 13,032 cells.

For initial testing, the two biosensor strains were grown in 2xYT medium (10 g/L yeast extract, 16 g/L tryptone, 5 g/L NaCl, distilled water) overnight at 37℃, 250 rpm in glass tubes containing 5 ml 2xYT with kanamycin (25 μg/ml, 2xYT-Kana). Pre-cultures were used to inoculate 5 ml 2xTY-Kana and grown for up to 48 h at 37℃, 250 rpm, with sampling every 24 h to monitor growth and green fluorescence using a plate reader.

#### Growth-based biosensor in Escherichia coli

A previous publication was used as a basis to design the growth-based biosensor^41^. The *E. coli* BW25113 Δ(*alaA*, *alaC*, *avtA*; Ala-, alanine negative strain) was generated starting from an *E. coli* strain available that does not grow on glucose due to knockouts in several genes involved in glucose metabolism. In order to achieve the desired knockouts, we applied a described procedure^42^, which relies on the λ red recombineering system. The genes were knocked out sequentially following the same workflow: first the target gene was replaced with the *kana* resistance gene (also used for selection of positive clones) due to the controlled expression of the λ red recombinase, and the resistance cassette was then eliminated via the expression of the FLP recombinase. Both enzymes were transiently expressed in *E. coli* via a two plasmid-base system and both vectors could be easily cured as they harbor temperature-sensitive origin of replications. Each step was controlled via colony PCR to confirm the insertion of the antibiotic cassette and then its subsequent removal.

To allow the conversion of GABA into alanine, the strain was transformed with a plasmid harboring the *GABA-T* gene from *Arabidopsis thaliana*. The vector originated from a plasmid available in our collection (pPPro4) harboring the *sfGFP* and *mCherry* genes under the control of two copies of the same constitutive promoter in opposite directions. The *GABA-T* gene was ordered as a synthetic fragment (Twist Biosciences) without adapters, having additional 20 bp upstream and downstream the gene overlapping defined sequences in the vector upstream and downstream the *mCherry* gene. The plasmid was linearized via restriction digestion with *EcoRI* and *SphI* and the insert cloned using Gibson assembly. The mix was transformed in chemically competent DH10B *E. coli* cells and the positive transformants were confirmed by sequencing. The generated plasmid (pPPro_4) was then transformed into chemically competent *E. coli* Ala- cells.

The biosensor was then optimized to improve its sensitivity. The GABA-T gene was amplified using primers PP15 and PP16 and as template the synthetic DNA fragment used to generate pPPro_4 construct. The PCR product was cloned into a derivative of the pPPro_4 vector backbone which harbors a weak ribosome binding site (RBS) upstream of the *mCherry* gene (weaker than the RBS sequence used upstream the *GABA-T* gene in pPPro_4). The cloning was performed as described for the construction of pPPro_4, having the vector backbone linearized via digestion with *EcoRI* and *SphI* and the insert ligated via Gibson assembly. Finally, the construct (pPPro_5) was introduced into the Ala- strain by chemical transformation.

As both plasmids pPPro_4 and pPPro_5 contain the *sfGFP* gene under a constitutive promoter, the strains can synthesize both *GABA-T* and *sfGFP*. To ensure proper GABA uptake, a construct harboring the GABA transporter (synthesized from the *GabP* gene) was transformed in both biosensors. The *GabP* gene from *E. coli* was amplified with the primers PP17 and PP18 using as template a construct already available in our collection (from the shuttle vector pPPro1). The PCR product was cloned into a low copy plasmid (pPPro6) by restriction digestion using the enzymes *KpnI* and *EcoRI*, followed by ligation and electroporation into electrocompetent *E. coli* Ala- cells.

Testing of the different growth-based biosensors was performed in 96 well plates using M9 minimal medium. First the strains were grown overnight in glass tubes with 5 ml LB with kanamycin (final concentration 50 μg/ml; 37℃, 250 rpm). Then the cultures were diluted 1:100 in M9 medium supplemented with either different concentrations of GABA (0—20 mM) or 2 mM alanine as a positive control. The cells were grown in the plate reader at 37℃, monitoring growth and green fluorescence every 30 min for up to 66 h.

### Production and incubation of the nanoliter-reactors (NLRs)

GABA-producing strains and the two biosensors were encapsulated in alginate beads (i.e. NLRs) using the following procedure per batch: 8.0 g of 2.5% aqueous sterile filtered sodium alginate were mixed with 2 ml of 0.9% NaCl containing the cells to be encapsulated, and gently mixed for 1 min. The final mixture was transferred to a 24 ml syringe and processed through a laminar jet break up system (NISCO Engineering) equipped with a coaxial nozzle (diameter = 0.350 mm). The syringe pump was set at a flow rate of 2.0 ml min-1, the encapsulator frequency at 3.5 kHz and a constant air pressure of 42 mbar was applied. The alginate droplets produced by this set-up were collected in a magnetically stirred hardening solution (100 ml of 100 mM CaCl2, 20 mM MES pH 6, 0.05% cysteine) and allowed to harden for 20 min resulting in NLRs with a diameter of ~ 200 μm (volume ~ 4.5 nL).

The cells added to the 0.9% NaCl stock originated from glycerol stocks of liquid cultures in rich media. The producer cells were cultivated for up to 3 days in 12 ml MRS medium, while the sensor cells were grown in 5 ml LB medium overnight. Glycerol was added to a final concentration of 20% and 15% for the producer and the sensor cells, respectively. OD_600_ was then measured to have an estimate of the number of cells per ml of the glxcerol stocks. Before the encapsulation process, the glycerol stocks were gently thawed in ice. The number of bacterial cells to be included was defined according to the objective of the experiment and to achieve an average occupation between 0.2 and 1 producer cells per NLR (i.e. 2.1 × 10^5^ or 1.05 × 10^6^ cells per batch respectively) or 200 sensor cells per NLR (i.e. 2.1 × 10^8^ cells per batch).

After the 20 min hardening step, the NLRs were retained using a cell strainer (100 μm pore size, Corning) and washed once with a washing solution (100 ml of 10 mM CaCl2, 10 mM MES pH6, 0.05% cysteine). The encapsulation procedure was optimized as much as possible to reduce oxygen exposure.

Initially, it was planned to cultivate the three producer strains and biosensors in liquid culture, which was an- or microaerobically in SHIME medium (1 g/L complex sugars 5X, 2 g/L Hemin, 1 g/L vitamin mix 1000X, 8 g/L NaCl, 4 g/L K2HPO4, 4 g/L KH2PO4, 390 mg/L MgSO4, 220 mg/L CaCl2, 4 g/L MES, 1 g/L amino acid mix 10X, 1 g/L Cas alkaline mix 50X, 0.24 mg/L biotin, 11 mg/L galactose, distilled water). However, the high turbidity of the SHIME medium limited biomass detection. Therefore, the strains were grown directly in the NLRs and biomass formation was detected directly in the hydrogel carriers. The NLRs were collected in a sieve, and 1 g was used for further testing for growth in the NLRs, and under gut-like conditions. For these two testing conditions, alginate NLRs were weighed into the cultivation vessel at a concentration of 100 g/L and incubated in medium according to Table 2.

### COPAS (Complex parametric analyzer and sorter) analysis and sorting of NLRs

The NLRs were analyzed and sorted by a COPAS Biosorter (Union Biometrica, Holliston, MA, USA), which can analyze, sort, and dispense objects ranging from 20—500 microns in size by using the object length, the optical density and the intensity of fluorescent signals. In the configuration for this project the COPAS had three excitation lasers (405 nm, 488 nm, 651 nm) and three emission band filters (445/40 nm, 545/25 and 615/24 nm) to detect the fluorescence of the blue, green and red regions of the spectrum. The 488 nm laser was used to measure the optical density of the objects. The NLRs in the sheath fluid are passed through a flow cell, where they are illuminated by the lasers and the optical extinction (optical density of the detected object) as well as simultaneous quantification of the fluorescence signals (blue, green and red) can be performed.

### Microscopy

Pictures were taken using a Zeiss Axio Observer 1. Images in the report are either labeled as bright field images (BF) or fluorescent light images (GFP (excitation band pass 470/40 nm; beam splitter 495 nm; emission band pass 525/50 nm) or RFP (excitation band pass 565/30 nm; beam splitter 585 nm; emission band pass 620/60 nm) filter sets) or as multi-channel overlays (eg. BF/GFP or BF/GFP/RFP).

### Statistical analysis

Data were analysed and graphs were produced using GraphPad Prism 10 for Windows (GraphPad Software LLC). Means were compared using two-sided unpaired t-tests and statistical significance was assumed when *p* < 0.05.

### Supplementary Information

Supplementary Information.

## Data Availability

Proprietary dsm-firmenich strains may be made available for academic research under a Material Transfer Agreement with dsm-firmenich. The datasets generated during the current study are available in the OSF repository, https://osf.io/tvafn/?view_only=1e8c35ac10d04c8bb99091c2207622a1.
