# Supplementary material for: Development of a workflow for the selection, identification and optimization of lactic acid bacteria with high γ-aminobutyric acid production
Source: Sci Rep. 2023 Aug 22;13:13663. doi: 10.1038/s41598-023-40808-z (PMC10444875; doi:10.1038/s41598-023-40808-z)
Supplement: Supplementary file 1 — Supplementary Information. [file 41598_2023_40808_MOESM1_ESM.pdf]

Supplementary information for manuscript:

Development of a workflow for the selection, identification and optimization of lactic acid bacteria with high γ-aminobutyric acid production

Ateequr Rehman, Giulio Di Benedetto, Julia K. Bird, Valentina Dabene, Lisa Vadakumchery, Ali May, Ghislain Schyns, Wilbert Sybesma, Tim N. Mak.

Supplementary table S1: Primer sequences

Primer	Sequence 5'-3'
PP9	AGAGTTTGATCCTGGCTCAG
PP10	AAGGAGGTGATCCAGCCGCA
PP11	AAGTGCCATTCCGCCTGA
PP12	ATATGCATGCATGGTCGTTATCAATTCG
PP13	CGAATTGATAACGACCATGCGTATACCTCCTCATTAAAGTTAAACAAAATTATG
PP14	ATGGTCGTTATCAATTCGCTGCGTCGTCGTGC
PP15	AGGCCGCCTAGGCCGCGGCCGCGCGTTATTATTTTTTGTGCTGGGCTTTCAGTTC
PP16	GTTTAACTTTAAGAAGGAGATATACGATGGTCGTTATCAATTCGCTGCG
PP17	ATATGGTACCGAGCTCTTTACAGCTAGCTCAGTCCTA
PP18	ATATGAATTCCTTAGCGCGTATTATGAAC

Supplementary table S2: Plasmid characteristics

Plasmid	Characteristics (origin, resistance, genes)
pPPro1	colE1, Kanamycin, GabR
pPPro2	pHmI519, Kanamycin, sfGFP
pPPro3	pHmI519, Kanamycin, GabR, sfGFP
pPPro4	p15A, Kanamycin, GABAT
pPPro5	p15A, Kanamycin, GABAT
pPPro6	pSC101, Ampicillin, GabP
